# Microbial Community Changes in a Chlorinated Solvents Polluted Aquifer Over the Field Scale Treatment With Poly-3-Hydroxybutyrate as Amendment

**DOI:** 10.3389/fmicb.2018.01664

**Published:** 2018-07-24

**Authors:** Bruna Matturro, Lucia Pierro, Emanuela Frascadore, Marco Petrangeli Papini, Simona Rossetti

**Affiliations:** ^1^Water Research Institute, IRSA–CNR, Rome, Italy; ^2^Department of Chemistry, Sapienza University of Rome, Rome, Italy

**Keywords:** reductive dehalogenation, *D. mccartyi*, PHB, reductive dehalogenase genes, groundwater circulation wells, bioremediation

## Abstract

This study investigated the organohalide-respiring bacteria (OHRB) and the supporting microbial populations operating in a pilot scale plant employing poly-3-hydroxybutyrate (PHB), a biodegradable polymer produced by bacteria from waste streams, for the *in situ* bioremediation of groundwater contaminated by chlorinated solvents. The bioremediation was performed in ground treatment units, including PHB reactors as slow release source of electron donors, where groundwater extracted from the wells flows through before the re-infiltration to the low permeability zones of the aquifer. The coupling of the biological treatment with groundwater recirculation allowed to drastically reducing the contamination level and the remediation time by efficiently stimulating the growth of autochthonous OHRB and enhancing the mobilization of the pollutants. Quantitative PCR performed along the external treatment unit showed that the PHB reactor may efficiently act as an external incubator to growing *Dehalococcoides mccartyi*, known to be capable of fully converting chlorinated ethenes to innocuous end-products. The slow release source of electron donors for the bioremediation process allowed the establishment of a stable population of *D. mccartyi*, mainly carrying *bvcA* and *vcrA* genes which are implicated in the metabolic conversion of vinyl chloride to harmless ethene. Next generation sequencing was performed to analyze the phylogenetic diversity of the groundwater microbiome before and after the bioremediation treatment and allowed the identification of the microorganisms working closely with organohalide-respiring bacteria.

## Introduction

Over the past 20 years, intense research efforts have been devoted to elucidate the overall mechanisms underlying the biological reductive dechlorination (RD) process and to develop effective technologies for the *in situ* remediation of contaminated sites (Lorenz and Löffler, 2016).

The RD process is performed by specialized organohalide-respiring bacteria (OHRB) which enable the complete and efficient detoxification of a variety of aliphatic and aromatic chlorinated pollutants. Among the many bacterial species that are now known to reductively transform organohalides, *Dehalococcoides mccartyi* is considered as the biomarker of the process due to the unique ability of members of this genus to fully convert chlorinated solvents to harmless products through the activity of a class of enzymes called reductive dehalogenases (RDases) (Richardson, 2013). They are involved in the metabolic dechlorination of PCE or TCE to VC (TceA), of *cis*-DCE to VC and ethene (BvcA and VcrA) and are coded by the corresponding genes *tceA, bvcA*, and *vcrA* (Lee et al., 2006).

Recently, several site investigations showed the relevance of the geochemical monitoring integrated with the biomolecular analysis of both OHRB and flanking microbial communities (Kotik et al., 2013; Kao et al., 2016; Atashgahi et al., 2017). The use of an integrated monitoring approach may reduce uncertainties about the ongoing groundwater processes and allow an efficient long-term management of the remedial action (Majone et al., 2015).

*In situ* stimulation of native OHRB through the addition of fermentable substrates represents one of the main approaches used for remediating chlorinated solvents contaminated aquifers (Steffan and Schaefer, 2016). Recently, poly-β-hydroxy-butyrate (PHB) was shown to be effective as a slow-release electron donor for the RD process (Aulenta et al., 2008; Pierro et al., 2017). PHB is an inert, biocompatible and fully biodegradable material which has been proposed for several attractive biotechnological applications (Williams and Martin, 2005). It is a polyester synthesized as a carbon and energy reserve material by a wide number of prokaryotes. More than 300 species, mainly of bacteria, have been reported to produce these polymers (Olivera et al., 2001; Chanprateep, 2010; Centeno-Leija et al., 2014). PHB is industrially produced by microbial fermentation using bacterial strains, cultivated on inexpensive carbon sources such as beet and cane molasses, corn starch, alcohols, and vegetable oils (Lee, 1996; Chen, 2009, 2010; Chanprateep, 2010; Peña et al., 2011, 2014).

In order to act as slow-release electron donor, PHB is enzymatically hydrolyzed to 3-hydroxybutyrate (HB) which is then converted to acetate and H_2_ through β-oxidation.

To date, only a few laboratory studies investigated the efficacy of PHB as electron donor in the RD process (Aulenta et al., 2008; Baric et al., 2012, 2014) and the first pilot scale PHB application was only recently documented (Pierro et al., 2017). A combination of a groundwater circulation wells (GCWs) with an external treatment unit, including a PHB reactor allowing continuous delivering of electron donor in the contaminated aquifer, was installed at a chlorinated solvent contaminated aquifer where partial biological RD quantitatively transformed higher chlorinated ethenes and ethanes (utilized at the site in industrial degreasing operation) in the less chlorinated *cis-*DCE and VC.

The effectiveness of PHB as a suitable electron donor source for enhancing the *in situ* RD of *cis*-DCE to non-toxic ethene in low permeability contaminated aquifer was clearly demonstrated during the plant operation and the RD process was found to be mirrored by the occurrence of both *D. mccartyi* and reductive dehalogenase genes (Pierro et al., 2017).

As an unexpected effect of the continuous recirculation of the contaminated groundwater through the external treatment unit, the occurrence of a biological dechlorination activity was revealed, after around 200 days of operation, both in the PHB and zero valent iron (ZVI) reactors with the almost quantitative removal of the extracted *cis*-DCE and VC. The stimulation of the biological reductive activity resulted from the *in situ* enhancement of the RD by the GCW operation. By this regard, extracted groundwater, enriched with OHRB as result of the electron donor continuous amendment, passes through the external reactors in the treatment unit where dechlorinating microorganisms find the optimal growth conditions (reductive redox conditions and electron donor concentration in the PHB and ZVI reactors).

In this paper, we report the throughout investigation on the microbial changes and the behavior of OHRB along the external operation unit of the pilot plant, focusing in particular on the PHB reactor and on the structure and role of the microbial community involved in the RD process driven by the fermentation of this slow release carbon source.

## Materials and Methods

### Pilot Field Test

**FIGURE 1:**
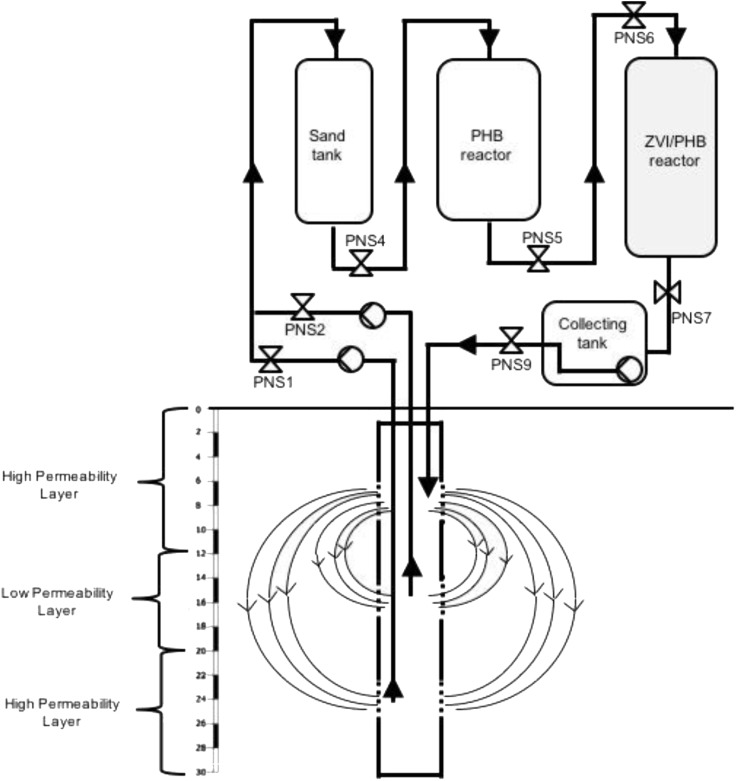
Schematic illustration showing the pilot plant (GCW and external treatment unit) and the position of the sampling valves (PN).

As described in Pierro et al. (2017), the process is carried out by extracting contaminated water through a multi-screened 30-m-deep IEG-GCW^®^ and transferring it to an external treatment unit which comprises a sand tank (for suspended solid filtration), a reactor containing the fermentable PHB polymer and one with ZVI for the removal of the extracted contaminants before reinjection. Valves were installed along the plant for sample collection and analyses (**Figure 1**). IEG-GCW^®^ plant was designed based on geological and hydrogeological conditions of the site and it consists in a single borehole screened at three different depths separated by packers (first permeable layer, low permeability intermediate layer, and deep permeable layer). Three centrifugal pumps were installed on the surface. Two pumps extracted groundwater from the deep permeable layer (from 22 to 26 m below the ground surface, bgs) and the low permeability intermediate layer, where a significant mass of chlorinated solvents was strongly retained by aging phenomena (from 15 to 19 m bgs). After passing through an external treatment unit, pumped groundwater is discharged back into the aquifer through the upper screened section (from 8 to 12 m bgs), thus creating two circulation groundwater cells (**Figure 1**). Groundwater recirculation by GCW coupled with electron donor continuous amendment (by the PHB reactor) effectively allowed chlorinated solvents mobilization and *in situ* RD stimulation in the low accessible heavily contaminated low permeable aquifer zones.

### Chemical Analysis

Chlorinated solvents were determined by headspace gas chromatographic analysis. Groundwater samples were collected directly from gate valves (PNS1-7) in 10 ml pyrex vials. Each vial was completely filled to leave almost no headspace, sealed with a Teflon-faced butyl septum and transported to the laboratory. In order to create headspace and to perform gas chromatographic analysis, 1 ml of each groundwater sample was transferred into a second 10 ml pyrex vial sealed with a Teflon-faced butyl septum. Headspace analysis of chlorinated compounds was performed using a gas chromatograph (Master DANI) equipped with a DANI 86.50 headspace autosampler. The chromatograph was fitted with a TRB 624 (75 m × 0.53 mm i.d) capillary column and a flame ionization detector (FID, 300°C). Sample injection was operated in splitless mode, where the injector temperature was set at 180°C. Helium was used as carrier gas at a constant flow of 10 mL min^-1^ and the GC oven was temperature programmed as follows: 50°C for 0.5 min increasing at 15°C min^-1^ to 180°C for 0 min then increasing at 40°C min^-1^ to 210°C for 0 min. The headspace autosampler conditions were: oven temperature 80°C, manifold temperature 120°C, transfer line temperature 180°C, shaking soft. The GC was previously calibrated with standard cis-DCE and VC concentrations over a linear response range.

### Sampling for Biomolecular Analysis

Samples for biomolecular analysis (500 mL of groundwater) were collected before the bioremediation treatment (*T* = 0) from the groundwater wells PNS1 (groundwater from high permeable zone with low concentration of *cis-*DCE and VC) and PNS2 (groundwater from the low permeable zone with high concentration of *cis-*DCE and VC). Water samples were taken also after 570 days of plant operation from PNS1, PNS2 and from sampling points located in the external unit of the pilot plant PNS4 (out of the sand tank), PNS5, and PNS6 (out of the PHB reactor unit), PNS7 (out of the ZVI/PHB reactor unit).

Each groundwater sample was filtered on polycarbonate filters (pore size 0.22 μm, 47 mm diameter, Nuclepore) to harvest the biomass and DNA was extracted by Power Soil DNA extraction kit (MoBio, Italy) following the manufacturer’s instructions. Purified DNA from each sample was eluted in 100 μL sterile Milli-Q and stored at -20°C until further analysis.

### Quantitative PCR

Quantitative PCR (qPCR) targeting *D. mccartyi* 16S rRNA genes and reductive dehalogenase genes *tceA, bvcA, vcrA* was conducted on DNA extracted from PNS1 and PNS2 before the bioremediation treatment and from the sampling points located in the external unit of the treatment plant (PNS1, PNS2, PNS4, PNS5, PNS7) after 570 days of operation. qPCR absolute quantification with TaqMan^®^ chemistry was applied. Reactions were performed in 20 μL total volume of SsoAdvanced^TM^ Universal Probes Supermix (Bio-Rad, Italy), including 3 μL of DNA as template, 300 nM of each primer and 300 nM of TaqMan^®^ probe composed by 6-carboxyfluorescein (FAM) as the 5′ end reporter fluorophore and *N*,*N*,*N*,*N*,-tetramethyl-6-carboxyrhodamine (TAMRA) as the 3′ end quencher. Primers and probes used for each reaction are listed in Supplementary Table S1. Standard curves for the absolute quantification were constructed by using the long amplicons method previously reported in Matturro et al. (2013). Each reaction was performed in triplicate with CFX96 Touch^TM^ Real-Time PCR Detection System (Bio-Rad, Italy). Quantitative data were expressed as gene copy numbers L^-1^ and error bars were calculated with Microsoft Excel^®^ on triplicate reactions for each sample.

### Next Generation Sequencing (NGS)

Next generation sequencing (NGS) was performed on groundwater samples collected at PNS1 and PNS2 before the PHB treatment (*T* = 0) and at the outlet of the PHB reactor (PNS5) after the bioremediation treatment (570 days of plant operation).

10 ng of DNA extracted from each groundwater sample (500 mL) was used for NGS analysis. 16S rRNA Amplicon Library Preparation (V1–3) was performed as detailed in Matturro et al. (2017). The procedure for bacterial 16S rRNA amplicon sequencing targeting the V1–3 variable regions is based on Caporaso et al. (2012), using primers adapted from the Human Gut Consortium (Ward et al., 2012). PCR reactions were performed in 25 μL reaction volume containing Phusion Master Mix High Fidelity (Thermo Fisher Scientific, United States) and 0.5 μM final concentration of the library adaptors with V1–3 primers (27F: 5′-AGAGTTTGATCCTGGCTCAG-3′; 534R: 5′-ATTACCGCGGCTGCTGG-3′). All PCR reactions were run in duplicate and pooled afterward. The amplicon libraries were purified using the Agencourt^®^ AMpure XP bead protocol (Beckmann Coulter, United States). Library concentration was measured with Qubit 3.0 fluorometer (Thermo Fisher Scientific, United States). The purified libraries were pooled in equimolar concentrations and diluted to 4 nM. 10% Phix control library was spiked in to overcome low complexity issue often observed with amplicon samples. The samples were paired end sequenced (2 × 301 bp) on a MiSeq (Illumina) using a MiSeq Reagent kit v3, 600 cycles (Illumina, United States) following the standard guidelines for preparing and loading samples on the MiSeq.

Next generation sequencing secondary data were processed and analyzed using QIIME2 software tools 2018.2 release (Caporaso et al., 2010). The reads were demultiplexed using demux plugin^1^, denoized, dereplicated and chimera-filtered using DADA2 algorithm (Callahan et al., 2016). The taxonomic analysis was based on a Naïve–Bayes classifier trained on 16S rRNA gene OTUs clustered at 99% similarities within the Silva 128 database (Quast et al., 2013). The alpha-diversity analysis was performed by PAST version 2.17 (Hammer et al., 2001) using total OTUs generated from each sample. Rarefaction curves were computed using the Vegan R package.

### 16S rRNA Gene Clone Library

A 16S rRNA gene clone library was obtained from DNA extracted at PNS5 sampling point after 570 days of plant operation. DNA was amplified using primers 27f (5′-AGAGTTTGATCMTGGCTCAG-3′) and 1492r (5′-TACGGYTACCTTGTTACGACTT-3′) using Hot Start Taq98 (Lucigen, Italy). PCR reactions were performed with the following cycles: 2 min at 98°C, 38 cycles for 30 s at 98°C, 30 s at 58°C, 1 min at 72°C and final 15 min at 72°C. PCR products were purified using the QIAquick^®^ PCR purification kit (Qiagen, Milan, Italy). Cloning of PCR products was carried out using pGEM-T Easy Vector System (Promega, Italy) into *Escherichia coli* JM109 competent cells (Promega, Italy) according to the manufacturer’s instructions. Positive inserts were amplified from recombinant plasmids obtained from white colonies by PCR using the sequencing primers T7f (5′-TAATACGACTCACTATAGGG-3′) and M13r (5′-TCACACAGGAAACAGCTATGAC-3′). PCR amplicons of 1,465 bp length were purified using the QIAquick PCR purification kit (Qiagen, Milan, Italy) and sequenced with primers 530F (5′-GTGCCAGCMGCCGCCG-3′) and 907R (5′-CCGTCAATTCMTTTRAGTTT-3′). A total of 100 clones were screened by PCR and multiple sequence alignments were performed with ClustalW2 to check the similarity among the sequences. The 16S rRNA gene sequences were deposited in the GenBank database under accession numbers from MG251533 to MG251561.

### Phylogenetic Analysis

The 16S rRNA sequences were analyzed with the ARB software (Ludwig et al., 2004) using the SILVA 16S rRNA SSU Reference database release 102 (Pruesse et al., 2007). Sequences were screened for chimeras using DECIPHER (Wright et al., 2012). The phylogenetic tree was constructed using the maximum likelihood method implemented in the program RAxML (Stamatakis et al., 2008). Bootstrap analyses were conducted using 1,000 resampling replicates. *Planctomycetes* was chosen as outgroup.

## Results

### External Treatment Unit Performances After 200 Days of Operation

**FIGURE 2:**
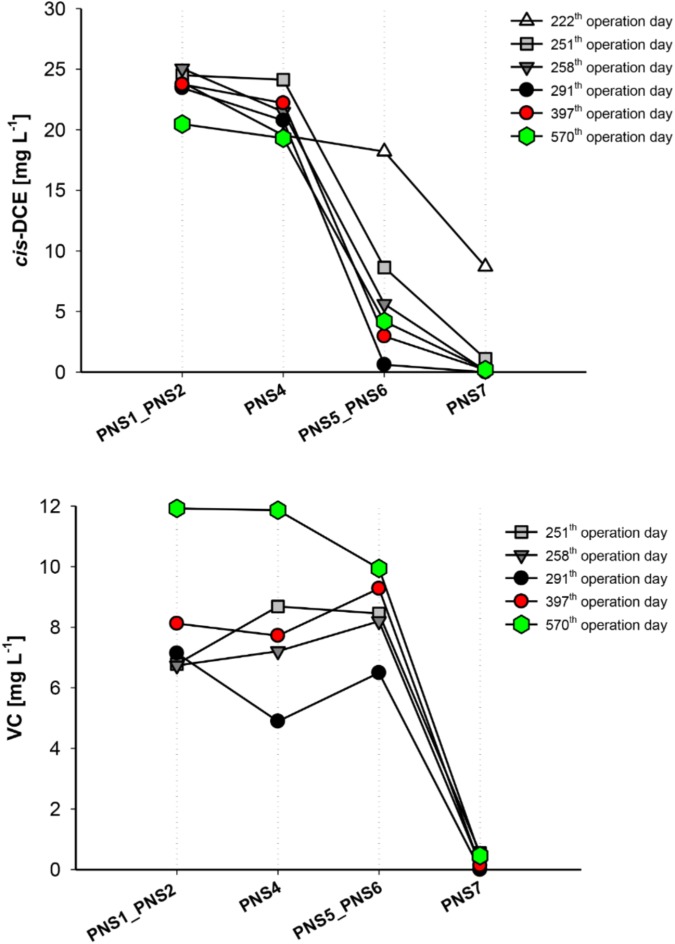
*cis*-DCE and VC reductive dechlorination at different sampling times over long-term plant operation. PNS1 and PNS2 average concentrations are shown.

Profiles of *cis*-DCE and VC concentration along the external treatment unit at different operation days are shown in **Figure 2**. After about 200 days of continuous operations, *ci*s-DCE concentration starts to decline in the PHB reactor and was almost quantitatively removed at the outlet of the ZVI/PHB reactor. VC concentration slightly increased in the PHB reactor and was then completely removed after passing through the ZVI/PHB reactor. Considering that no chemical reductants were present in the PHB reactor and that VC is not efficiently reduced by ZVI reactor, this behavior could be explained only by the occurrence of the biological RD of both *cis*-DCE and VC.

### *D. mccartyi* and Reductive Dehalogenase Genes Quantification

The biomarkers of RD process, including *D. mccartyi* 16S rRNA and functional RDase genes *tceA, bvcA*, and *vcrA*, were quantified by qPCR in the groundwater samples collected before the treatment and after 19 months of plant operation (*T* = 570 days) at different sampling points along the external unit of the pilot plant (PNS1, PNS2, PNS4, PNS5, PNS6, PNS7). As shown in Supplementary Figure S1, similar *D. mccartyi* abundances were found at PNS1 and PNS2 before treatment (2.24E + 06 and 4.02E + 06 16S rRNA gene copies L^-1^, respectively). They were mainly composed by *D. mccartyi* strains carrying *tceA* and *vcrA* (PNS1: 2.02E + 06 *tceA* gene copies L^-1^; 1.12E + 06 *vcrA* gene copies L^-1^; PNS2: 3.06E + 06 *tceA* gene copies L^-1^; 3.04E + 06 *vcrA* gene copies L^-1^).

At the end of the treatment, *D. mccartyi* increased in both samples (3.12E + 07 and 7E + 07 gene copies L^-1^ in PNS1 and PNS2, respectively) and remained quite unvaried after the groundwater flew through the inert sand tank PNS4 (6.10E + 07 gene copies L^-1^). Interestingly, *D. mccartyi* increased at the outlet of the PHB reactor (PNS5) where a total of 2.54E + 08 gene copies L^-1^ was found. At the sampling point located before the ZVI/PHB reactor (sampling point PNS6) *D. mccartyi* abundance was similar to PNS5 (2.49E + 08 gene copies L^-1^) and slightly decreased to 6.72E + 07 gene copies L^-1^ before the re-inoculation of the groundwater into the aquifer (sampling point PNS7).

**FIGURE 3:**
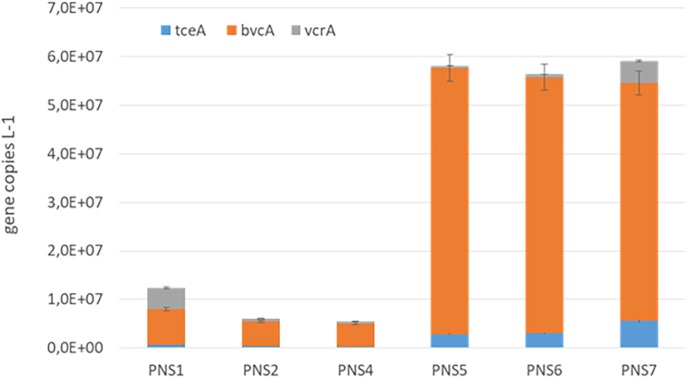
Quantification of *D. mccartyi* strains carrying *tceA, bvcA*, and *vcrA* reductive dehalogenase genes along the external units of the pilot plant at 570 days of the plant operation.

In line with chemical data, *D. mccartyi* strains carrying *bvcA* genes represented the main component with only a limited occurrence of *vcrA* and *tceA* genes along the all external treatment unit (**Figure 3**).

### Microbial Community Composition of the PHB Reactor

The impact of the treatment on the structure and composition of the groundwater indigenous microbial communities was assessed by 16S rRNA gene high-throughput sequencing.

The analysis performed on groundwater samples collected from PNS1, PNS2, and PNS5 yielded between 63,546 and 108,169 sequence reads after bioinformatic processing (Supplementary Table S2). Rarefaction curves showed that the reads obtained were sufficient to capture sample diversity (Supplementary Figure S2).

As shown in Supplementary Figure S3, the contaminated groundwater before plant operation was mainly composed by *Proteobacteria* (∼50% of total OTUs in PNS1 and ∼67% of total OTUs in PNS2) whereas members of *Chloroflexi* phylum represented max ∼2% of total OTUs in both groundwater samples. Interestingly, sequences affiliated to *Parcubacteria* were found at high abundance (∼22%) in the groundwater from high permeable zone with low concentration of chlorinated contaminants (PNS1). Members of *Parcubacteria* were recently identified by phylogenetic analysis of 16S rRNA genes recovered from environmental samples and metabolic predictions indicate that they have very restricted metabolic potential, largely based on fermentation (Castelle et al., 2017).

**FIGURE 4:**
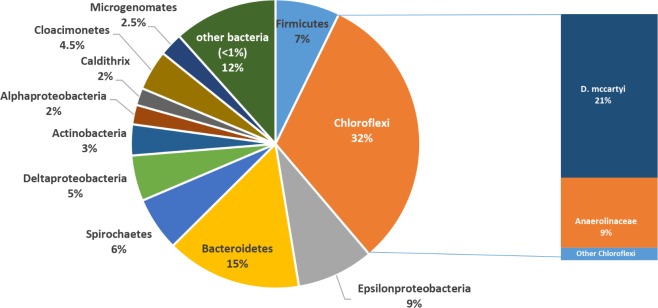
Microbial community structure of the treated groundwater (PNS5 sampling point) after long-term plant operation.

After long-term operation (570 days), the microbiome composition of groundwater drastically changed with the marked increase of *Chloroflexi* (**Figure 4**). In detail, *Chloroflexi* represented up to 32% of total OTUs and included members of *Dehalococcoidaceae* (21% of total OTUs, mostly composed by sequences affiliated to *D. mccartyi* genus) and *Anaerolinaceae* (9% of total OTUs, mainly *Leptolinea* and *Pelolinea* genera) families (**Figure 4**). Other phyla found in PNS5 were affiliated to *Proteobacteria* (20% of total OTUs, including 9% of *Epsilonproteobacteria*, and 5% of *Deltaproteobacteria*), *Bacteroidetes* (15%), *Firmicutes* (7%), *Spirochaetes* (6%), *Cloacimonetes* (5%), and *Actinobacteria* (3%). Low-abundance OTUs were affiliated to *Microgenomates* (2.5%), *Alphaproteobacteria* (2.0%), *Caldithrix* (2%) and to a variety of taxa occurring at <1% of total OTUs (∼12%) including sequences affiliated to poorly characterized bacterial lineages such as *Aminicenantes, Caldiserica, Parcubacteria*, and *Peregrinibacteria*.

### 16S rDNA Clone Library

**FIGURE 5:**
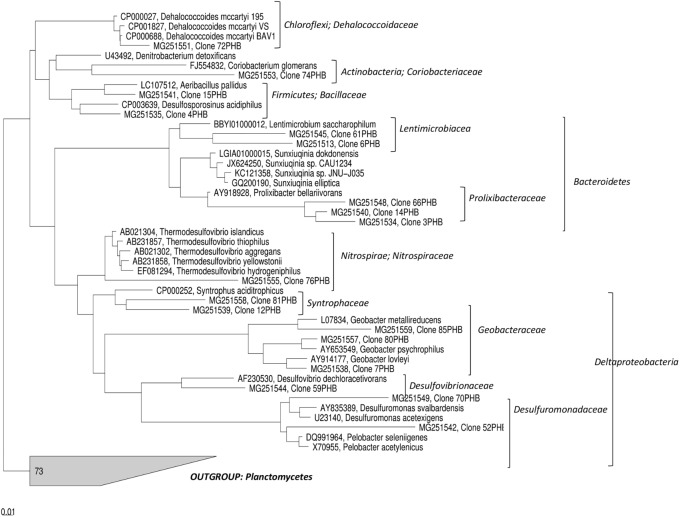
Phylogenetic tree of 16S rRNA gene sequences obtained from the clone library constructed with DNA extracted from PNS5 sampling point after 570 days of treatment.

The characterization of the microbial communities selected in the PHB reactor was further performed by constructing 16S rDNA clone library to recover near-full-length 16S rRNA gene sequences. A total of 100 clones containing inserts with the expected size (about 1.5 kb) were selected from the total library, partially sequenced and sequence identities determined (**Table 1**). Partial sequences differing by ≤2% were considered a single relatedness group and representatives of most of these groups were fully sequenced on both strands. **Figure 5** shows the phylogenetic tree of representative full-length sequences in the contest of currently recognized bacterial phyla. The analysis of the sequences showed the occurrence of microorganisms mostly with low 16S rRNA gene similarity (<90%) to already previously identified and/or cultured neighbors (**Figure 5**). The highest number of the screened clones belonged to *Lentimicrobiaceae* (20%) and *Nitrospiraceae* (20%) families followed by *Dehalococcoidaceae* (15%), *Prolixibacteraceae* (14%), *Syntrophaceae* (8%), *Coriobacteriaceae* (6%), *Desulfovibrionaceae* (4%) and *Desulfuromonadaceae* (4%) families.

**Table 1:** Numbers of the screened clones and the corresponding taxonomic affiliation.

No. of clones (total clones: 100)	Fully sequenced clones	Taxonomic affiliation
20	Two clones (6PHB, 61PHB)	Bacteroidetes; *Lentimicrobiaceae*
20	Clone 76PHB	Nitrospirae; *Nitrospiraceae*
14	Three clones (14PHB, 66PHB, 3PHB)	Bacteroidetes; *Prolixibacteraceae*
13	Three clones (85PHB, 80PHB, 7PHB)	Deltaproteobacteria; *Geobacteraceae*
8	Two clones (12PHB, 81PHB)	Deltaproteobacteria; *Syntrophaceaea*
6	Clone: 74PHB	Actinobacteria; *Coriobacteriaceae*
5	Clone: 15PHB	Firmicutes; *Bacillaceae*
1	Clone: 4PHB	Firmicutes; *Peptococcaceae*
5	Clone: 72PHB	Chloroflexi; *Dehalococcoidaceae*
4	Clone: 59PHB	Deltaproteobacteria; *Desulfovibrionaceae*
4	Clones: 52PHB and 70PHB	Deltaproteobacteria; *Desulfuromonadaceae*


In addition, other bacterial taxa present in decreasing abundance were *Geobacter* species, *Dehalococcoides mccartyi*, and *Aeribacillus pallidus* (**Table 1**).

## Discussion

The reliable and robust application of bioremediation strategies requires the comprehension of the microorganisms responsible for the contaminant degradation as well as the role of the flanking microbial communities. The detailed description of the groundwater microbial community able to reduce chlorinated solvents by using PHB as a slow-release electron donor source is reported in this study. The pilot plant was installed in a site heavily contaminated by *cis-*DCE and was composed by a GCW system connected to an external treatment unit where a PHB tank was installed to deliver fermentation products directly through the low permeable zones and improve the distribution of soluble electron donors. This coupled system allowed to create an effective three-dimensional circulation cell in the aquifer reaching less permeable layers where significant masses of contaminants are potentially accumulated (Pierro et al., 2017).

Overall, the treatment changed the groundwater microbiome with the remarkable increase of specialized OHRB able to dechlorinate *cis-*DCE and VC to ethene. This finding is in line with the evidences obtained from a preliminary laboratory treatability study, performed on soil core and groundwater samples taken from the same site, which indicated PHB as an effective source of electron donor able to stimulate the RD until the formation of ethene via transient VC formation (Pierro et al., 2017).

The analysis conducted along the external unit of the pilot plant employing PHB for the biological RD has demonstrated that the PHB reactor may efficiently act as an external incubator to growing *D. mccartyi*. Indeed, a marked increase of *D. mccartyi* strains, able to dechlorinate VC metabolically and more efficiently produce ethene, was found in the outlet streams of the PHB and ZVI/PHB reactors.

Additionally, the treatment stimulated the growth of a wide range of putative fermentative bacteria. High throughput sequencing revealed the occurrence of sequences belonging to *Leptolinea* genus. *L. tardivitalis*, the sole isolate available so far, is an obligate anaerobe able to ferment sugars and proteinaceous carbon sources and produce as by-products a variety of volatile fatty acids and hydrogen (Yamada et al., 2006). Other *Chloroflexi* found after the PHB treatment were affiliated to *Pelolinea*, previously described to have several phenotypic traits in common with members of the class *Anaerolineae*, e.g., strictly anaerobic growth and chemo-organotrophic metabolism with sugars and polysaccharides (Imachi et al., 2014). Additionally, sequences belonging to *Bacteroidetes* phylum were found at high abundance by NGS (∼15% of total OTUs) mainly affiliated to *Lentimicrobiaceae.* The further analysis of the near-full-length 16S rRNA gene sequences, generated by the clonal analysis, showed that most of them have a low similarity to already previously identified and/or cultured neighbors (≤90% 16S rRNA gene similarity). Among others, the screened clones were indeed mostly affiliated to *Lentimicrobiaceae* and *Prolixibacteraceae* (*Bacteroidetes* phylum) (**Table 1**). *Lentimicrobiaceae* family contains strictly anaerobic and slow-growing bacteria able to ferment a wide range of compounds to acetate, malate, propionate, formate and hydrogen (Sun et al., 2016). Within this family, *Lentimicrobium saccharophilum*, an anaerobic bacterium isolated from a reactor treating high-strength starch-based organic wastewater (Sun et al., 2016), displayed the highest sequence similarity (89–90%) with clones 6PHB and 61PHB. The sequences belonging to *Prolixibacteraceae* shared low levels of 16S rDNA sequence similarity (<90%) to known members of this family such as *Sunxiuqinia*, retrieved in anaerobic deep-sea environments (Qu et al., 2011) or *Prolixibacter*, fermentative bacterium isolated from a marine-sediment fuel cell (Holmes et al., 2007a), suggesting that they may belong to a novel taxonomic group at the genus level.

Additionally, a variety of sequences falling into taxonomic groups containing known sulfate- and Fe(III)- reducing bacteria was retrieved by the clonal analysis. They include members of *Nitrospiraceae*, mostly affiliated to the genus *Thermodesulfovibrio* (20% of the screened clones), and *Syntrophaceaea* family sharing 93–94% of sequence similarity with *Syntrophus aciditrophicus*, a fermenting bacterium able to grow on benzoate and certain fatty acids alone or in syntrophic association with hydrogen-consuming microorganisms (Elshahed and McInerney, 2001; Mouttaki et al., 2007). Furthermore, sequences related to *Denitrobacterium* genus (*Coriobacteriaceae* family) were found. This genus comprises a single species, *D. detoxificans*, able to grow via anaerobic respiration oxidizing hydrogen, formate or lactate for reduction of various oxidized nitrogen compounds (Anderson et al., 2000). Both NGS and clonal analysis revealed the occurrence of *Geobacteraceae* often found dominating in iron-reducing settings particularly in environments affected by anthropogenic influences (Holmes et al., 2007b). The *Geobacter* genus comprises anaerobic, non-fermenting chemoorganotrophic microorganisms able to reduce insoluble Fe(III) and Mn(IV) by employing several mechanisms including extracellular electron transfer via electric conductive nanowires. Being *Geobacter* species physiologically versatile, they are employed in environmental biotechnology, such as the natural attenuation of organic matter, bioremediation of aromatic hydrocarbons, heavy metals and organohalides, and generating bioenergy in microbial fuel cells and microbial electrolysis cells (Holmes et al., 2007b).

To lesser extent (4% of total clone library), sequences affiliated to *Desulfovibrionaceae* family were found sharing 97% of 16S rRNA sequence similarity with *Desulfovibrio dechloroacetivorans*, a microorganism able to coupling acetate oxidation to RD for growth (Sun et al., 2000). This group comprises strictly anaerobic members with a respiratory type or fermentative type of metabolism and most species are chemoorganoheterotrophs. Additionally, the analysis revealed the occurrence of sequences affiliated to *Desulfuromonadaceae* family, whose closest relatives were members of *Desulfuromonas* genus, such as *Desulfuromonas svalbardensis* able to reduce Fe(III) using common fermentation products such as acetate, lactate, propionate, formate or hydrogen as electron donor*s* (Vandieken et al., 2006), and *Pelobacter* genus including microorganisms with the ability to grow fermentatively on short-chain organic acids such as lactate, citrate and pyruvate such as *Pelobacter seleniigenes* (Narasingarao and Häggblom, 2007).

It is worth noting the occurrence of low-abundance OTUs affiliated to poorly characterized bacterial lineages such as *Aminicenantes, Microgenomates*, and *Cloacimonetes* phyla previously found in a wide variety of anaerobic environments including oil reservoirs and hydrocarbon-impacted sites (Farag et al., 2014; Hu et al., 2016; Stolze et al., 2017).

## Conclusion

The study confirmed the efficacy of the technology to remediate aquifers contaminated by chloroethenes and gave access the less represented species of the contaminated aquifer likely interacting with dechlorinators for the removal of chlorinated compounds through a wide range of metabolic reactions driven by the availability of PHB fermentation by-products. The results of this work clearly sustain the use of PHB as environmentally sustainable source of electron donor for bioremediation treatment of sites contaminated by chlorinated compounds and indicate the importance to further gain more insights into the metabolism networks of groundwater bacteria stimulated by the presence of PHB for the biological RD.

## Author Contributions

BM conducted qPCR, pyrosequencing, and analyzed the whole set of biological data. EF performed the bacterial 16S rRNA gene clonal analysis. LP performed the chemical analysis of the water samples and contributed to the interpretation of chemical data. SR and MPP conceived and coordinated the study. All authors contributed to the writing of the manuscript.

## Conflict of Interest Statement

The authors declare that the research was conducted in the absence of any commercial or financial relationships that could be construed as a potential conflict of interest.

## Footnotes

1 https://github.com/qiime2/q2-demux
