# Supplementary material for: Microbial Community Changes in a Chlorinated Solvents Polluted Aquifer Over the Field Scale Treatment With Poly-3-Hydroxybutyrate as Amendment
Source: Front Microbiol. 2018 Jul 24;9:1664. doi: 10.3389/fmicb.2018.01664 (PMC6066499; doi:10.3389/fmicb.2018.01664)
Supplement: Supplementary file 1 [file Presentation_1.pdf]

Supplementary Material

Microbial community changes in a chlorinated solvents polluted aquifer over the field scale treatment with poly-3-hydroxybutyrate as amendment

Matturro B.[1], Pierro L. [2], Frascadore E. [1], Petrangeli Papini M.[2], Rossetti S. [1]

[1] Water Research Institute, IRSA-CNR, Monterotondo, (RM), Italy

[2] Department of Chemistry, Sapienza University of Rome, Italy

Table S1. Primers and probes used for qPCR reactions.

Primers or probe	qPCR chemistry	Target gene	Reference
**Dhc 1200F: 5'-CTGGAGCTAATCCCCAAAGCT-3'**<br>**Dhc 1271R: 5'-CAACTTCATGCAGGCGGG-3'**<br>**Dhc probe: 5'FAM-TCCTCAGTTCGGATTGCAGGCTGAA-3'TAMRA**	TaqMan®	16S rRNA	Ritalahti et al., 2006*
**tceA 1270F: 5'-ATCCAGATTATGACCCTGGTGAA-3'**<br>**tceA1336R: 5'-GCGGCATATATTAGGGCATCTT-3'**<br>**tceA probe: 5'FAM-TGGGCTATGGCGACCGCAGG-3'TAMRA**	TaqMan®	tceA	Ritalahti et al., 2006
**bvcA 925F:5'-AAAAGCACTTGGCTATCAAGGAC-3'**<br>**bvcA 1017R: 5'CCAAAAGCACCACCAGGTC-3'**<br>**bvcA probe: 5'FAM-TGGTGGCGACGTGGCTATGTGG-3'TAMRA**	TaqMan®	bvcA	Ritalahti et al., 2006
**vcrA 1022F: 5'CGGGCGGATGCACTATTTT-3'**<br>**vcrA 1093R: 5'GAATAGTCCGTGCCCTTCCTC-3'**<br>**vcrA probe: 5'FAM-CGCAGTAACTCAACCATTTCCTGGTAGTGG-3'TAMRA**	TaqMan®	vcrA	Ritalahti et al., 2006

* Ritalahti, K.M., Amos, B. K., Sung, Y., Wu, Q., Koenigsberg, S.S., Löffler, F. E. (2006). Quantitative PCR Targeting 16S rRNA and Reductive Dehalogenase Genes Simultaneously Monitors Multiple *Dehalococcoides* Strains. Appl. Environ. Microbiol. 72(4), 2765–2774. doi: 10.1128/AEM.72.4.2765-2774.2006.

Table S2. Number of OTUs/reads and alpha-diversity indices for PNS1 and PNS2 (T=0) and PNS5 (T=570 days) samples.

	**PNS1**	**PNS2**	**PNS5**
OTUs	357	724	358
Reads	101235	108169	63546
Dominance_D	0,02	0,04	0,03
Simpson_1-D	0,98	0,96	0,97
Shannon_H	4,82	4,53	4,19
Evenness_e^H/S	0,35	0,13	0,18
Equitability_J	0,82	0,69	0,71
Chao-1	357	724	358,9

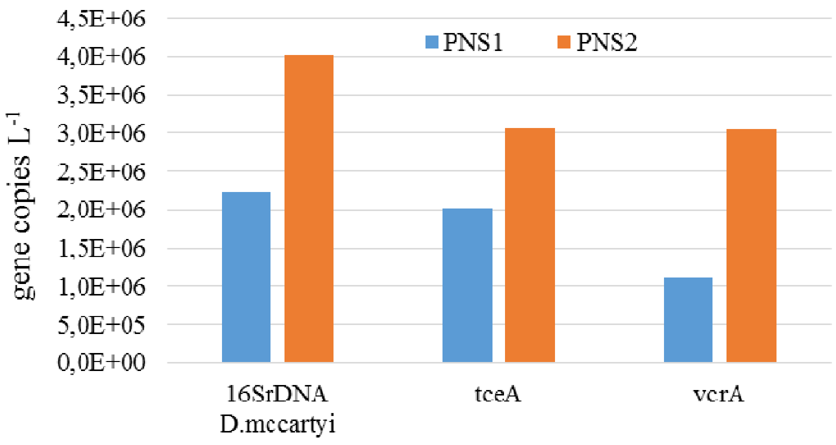

Figure S1. Quantification of *D. mccartyi* 16S rRNA genes and *tceA*, and *vcrA* reductive dehalogenase genes in groundwater samples collected from PNS1 and PNS2 before plant operation (T=0).

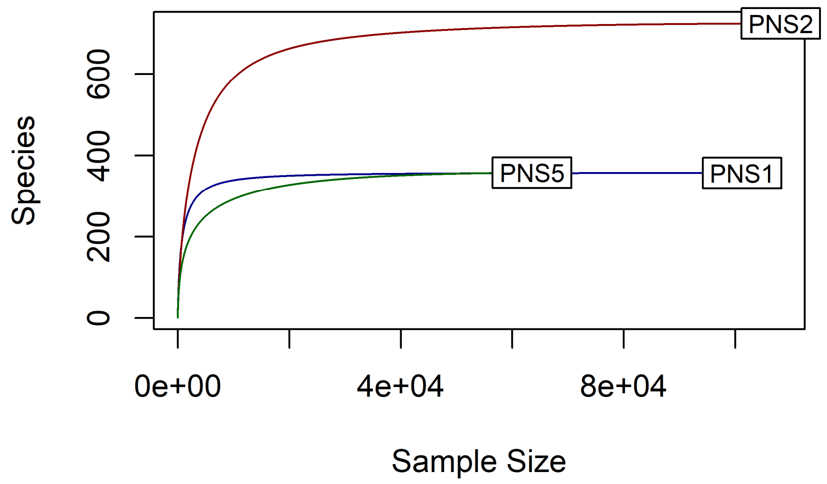

Figure S2. Rarefaction curves for PNS1, PNS2 and PNS5 groundwater samples.

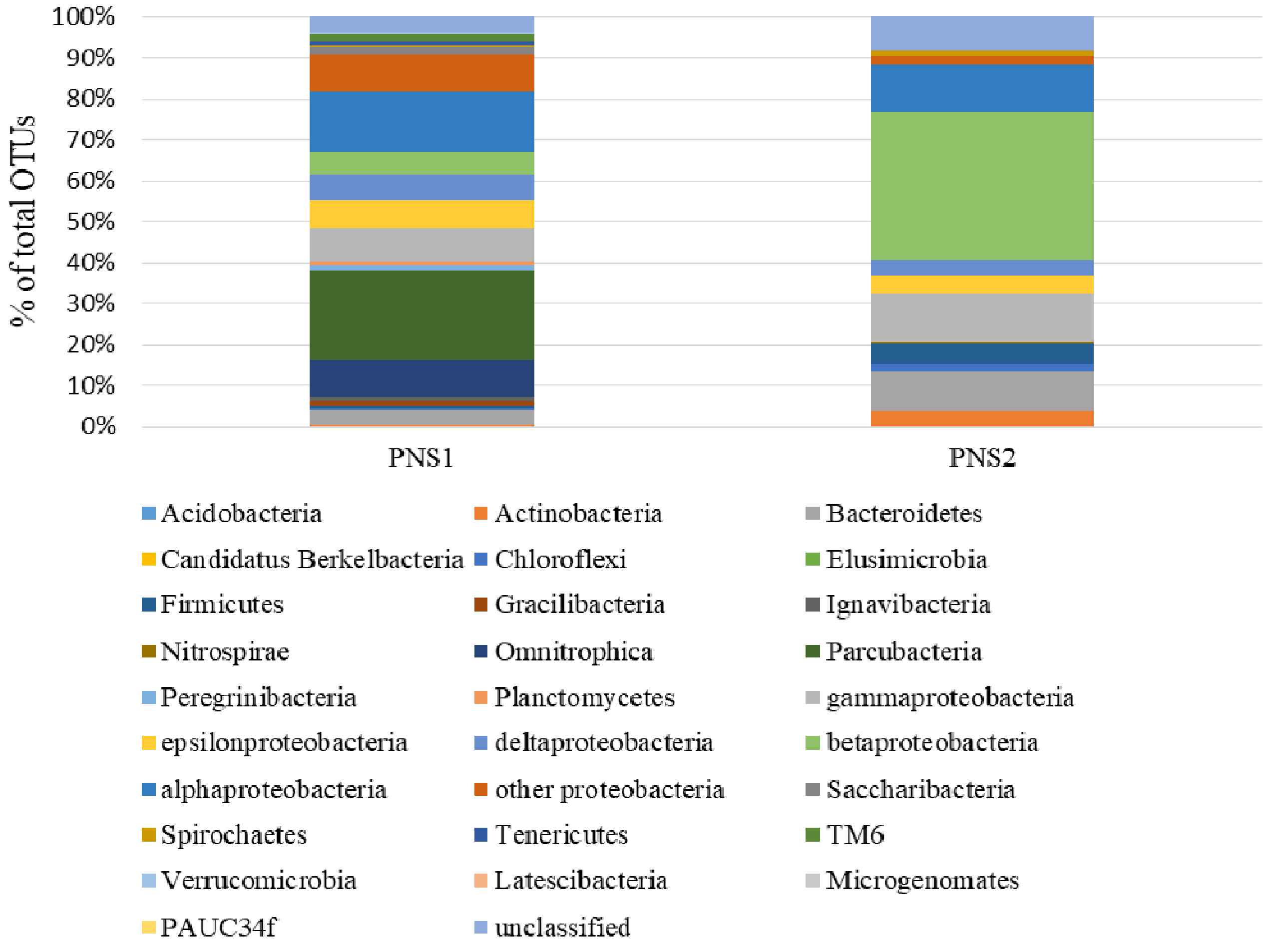

Figure S3. Microbial community structure of groundwater collected from PNS1 and PNS2 before plant operation (T=0).
